# “If that goes away, the house of cards falls”: challenges and solutions to ensuring CHW sustainability

**DOI:** 10.3389/fpubh.2026.1747834

**Published:** 2026-05-12

**Authors:** Ada M. Wilkinson-Lee, Dana R. Hunter, Katie C. Stalker, Nury Stemple, Flavio Marsiglia, Mary-Ellen Brown

**Affiliations:** 1 Department of Mexican American Studies, University of Arizona, Tucson, AZ, United States; 2 School of Social Work, The University of Texas at Austin, Austin, TX, United States; 3 School of Social Work, University at Buffalo, Buffalo, NY, United States; 4 School of Social Work-Watts College, Arizona State University, Tempe, AZ, United States

**Keywords:** CCR initiative, CHW workforce sustainability, community health workers (CHWs), Public Health Program Capacity for Sustainability Framework, qualitative participatory case study

## Abstract

The Community Health Workers (CHWs) for COVID Response and Resilient Communities (CCR) project supported CHWs to strengthen community resilience to combat COVID-19 and address health disparities. This qualitative, participatory case study aimed to identify the sustainability challenges and solutions faced by health organizations and care teams integrating CHWs as change agents. From June to August 2024, 27 interviews were conducted with CHWs (*n* = 9), program staff (*n* = 9), and program partners (*n* = 9), each having expertise in CHW workforce capacity. This study targeted two domains (funding stability and strategic planning) of the Public Health Program Capacity for Sustainability Framework. Data were analyzed using thematic analysis. Funding stability coalesced around three sub-themes: (1) *barriers to funding stability*, (2) *solutions to overcoming funding instability*, and (3) *the cascading impact on CHWs and communities*. Strategic planning was categorized into two prominent sub-themes: (1) the *systematic development of organizational strategic plans* and (2) *the use of data to support action-driven approaches* for ensuring the sustainability of the CHW workforce within and across organizations. The findings provide strategies and actionable steps that organizations need to take to fully sustain the CHW workforce within their health systems, including: full inclusion of CHW participation in every decision-making phase; the inclusion of CHW sustainability plans within organizational strategic plans; and rigorous participatory evaluations of CHW-led interventions to demonstrate how CHWs are making an impact on health disparities and promoting health equity. The implementation of all or part of these strategies has important implications for health system transformation and advancing health equity.

## Introduction

The COVID-19 pandemic and how we, as a nation, responded have altered our history and specifically the landscape of responding to public health crises. One of the positive outcomes of the pandemic was that community health workers (CHWs) were recognized as critical health responders at national and global levels for the first time (1, 2). Public health and community-based participatory researchers have collaborated and disseminated data demonstrating the reach and impact of the CHW workforce, especially in historically under-resourced communities (3–5). CHWs serve in multiple essential roles, including, but not limited to, improving access and continuity of health insurance coverage, enhancing provider–patient communication, monitoring health status, monitoring treatment adherence, and linking individuals to health and social services (6–8). Additionally, due to the ways in which CHWs interact with individuals and organizations across various levels of the health and social welfare sectors, they can help generate both intervention-specific and community-wide social capital through their daily activities (9). Reviews of studies have demonstrated that CHW interventions can lead to improvements across healthcare, including improved population health, improved patient experience of care, and reduced costs. CHW interventions also hold promise for advancing health equity (10, 11) by addressing the social determinants of health within several contexts, including neighborhoods, social services, and community organizations, in addition to healthcare institutions such as hospitals or clinics (12).

States recognize the need for CHWs to meet the diverse needs of their growing ethnic minority populations, yet most U.S. states struggle with how to support and sustain a CHW workforce (13). Managed care organizations that enter into contracts with states to provide health services to Medicaid members are highly motivated to achieve two aims that CHWs are skilled at supporting: improving outcomes and reducing the cost of care (14). In addition to existing evidence highlighting the benefits of CHWs within health systems, there is mounting evidence for a national strategy to support the development of CHW-covered services, billing codes, and metrics for CHW integration across systems and teams within the healthcare sector (15).

Community Health Workers for COVID Response and Community Resilience (CCR) was a Centers for Disease Control and Prevention (CDC) program that funded 67 health-serving organizations across the United States to mobilize CHWs to decrease the impact of COVID-19, address health disparities, and promote community resilience. The present study is a component of a participatory evaluation of the national CCR program. As the national study progressed, our CHW colleagues on the evaluation team identified CHW sustainability as an area that needed examination. Specifically, the purpose of this qualitative case study was to assess CHW sustainability challenges and solutions among a subset of 7 of the 67 CCR recipient sites tasked with training and deploying CHWs to combat COVID-19 by addressing health disparities. This study aimed to address the following questions: (1) What are the barriers to sustaining a CHW workforce within organizations and care teams? and (2) What are the strategies for CHW sustainability in organizations and care teams?

## Methods

The CDC and its evaluation partners [Arizona State University (ASU) and Washington State Evaluation Partnership (WSEP)] were committed to a set of equity-focused principles that emphasized participatory evaluation and guided the national evaluation of the CCR program, which included this case study. These principles were drawn from several evaluation approaches, including utilization-focused, empowerment, transformative participatory, naturalistic, critical theoretical, equitable, culturally responsive, and decolonizing approaches (16). The CCR program efforts were driven by ensuring CHW input and leadership were centered in all evaluation aspects, including planning, data collection, analysis, interpretation, and application of findings. The evaluation partners were committed to ensuring CHW input at every stage of the work; CHWs were recruited and compensated either as full-time study action team positions, consultants or Subject Matter Experts, or as members of the ASU’s Community Action Board or WSEP’s CHW Council. For the present study, CHWs were trained as interviewers, engaged in writing the questions for each of the participant categories, vetted the questions with their respective CHW coalitions, and conducted and/or reviewed the coding, analysis, and dissemination of the findings.

From June to August 2024, 27 interviews were conducted with CHWs (*n* = 9), program staff (*n* = 9), and program partners (*n* = 9), each having expertise in CHW workforce capacity. The program staff and program partners who participated in these interviews could be characterized as CHW allies and champions. All 67 CCR recipient sites were invited to a webinar describing the study, and sites submitted an interest form. Site selection criteria included: sites with a focus on sustainability with a diverse representation of CHWs, CHRs, and Promotores (as); sites that incorporated sustainable financing models for CHW services; sites with 8 or more CHWs hired with CCR funds; and sites with a diversity in rural/urban settings and in the types of sustainability models demonstrated. The larger study targeted four domains (political support, funding stability, organizational capacity, and strategic planning) (17). This manuscript is intended to focus on two domains (funding stability and strategic planning) of the Public Health Program Capacity for Sustainability Framework (18). CHW evaluators emphasized that it was important to conduct this case study on sustainability, given the complexity of sustainable funding mechanisms and the lack of shared terminology. This case study included seven CCR recipient sites with varying degrees of focus: three sites consisted of the entire state health system, three focused on county health services, and one focused on a specific large urban city. The research team deliberated on collecting demographic data from interview participants but opted not to collect traditional demographic information, given that some of the sites had eight or fewer CHWs and were the only CCR site in the state, and, as such, the data gathered could be matched back to a particular CCR location. Therefore, when providing descriptors for quotes, we will state the job category of the person providing the quote and not provide gender, ethnicity, or age descriptors.

The 1-hour interviews with CHWs, program staff, and program partners were fully structured and conducted via one-on-one Zoom sessions. The interview questions differed slightly for CHWs, program staff, and program partners. Participants ranged in their level of prior interaction with the interviewers based on how often each participant engaged in national CCR evaluation meetings, webinars, and evaluation meetings with CDC program officers and evaluation team members. Participants were given the option to review their transcripts, but no one selected this option. Participants agreed to hear the study findings during CCR webinars. The questions focused on the domains of the Public Health Program Capacity for Sustainability Framework related to funding and the sustainability of CHW positions. They ranged from asking about challenges, strategies, or providing the participant with a specific scenario. Each participant category had slightly worded scenarios based on their project role (see Table 1 for the interview questions). Participants received a $45 gift card in appreciation for their time. The Arizona State University Institutional Review Board approved all study procedures.

**Table 1:** Interview questions for each of the study participant categories.

Participant category	Question
Program CHWs	How does your organization currently fund CHWs? Are there multiple funding sources? Are there funding opportunities available that your organization DOES NOT take advantage of?
	Have there been any challenges or opportunities that affected your organization’s ability to support CHWs?
	What could your program or your organization do better to support CHWs and retain them in their positions?
	Has your organization planned for CHW sustainability after CCR? {If yes} How has leadership championed CHW sustainability? {If no} How has leadership created obstacles to CHW sustainability? Do you know if your position will continue after the CCR program ends?
	Have you been involved in meetings or workgroups at your organization to plan for CHW sustainability after CCR ends? {If yes} Please describe how you have been involved in this process. {If no} Please describe how you would like to be involved in this process.
Program staff	In an ideal world that does not have funding restrictions or other organizational limitations, what would the ideal CHW model within your organization look like?
	What do you think, as a supervisor/organization, are some of the issues or conditions that are holding back long-term employment of CHWs?
	What will it take for CHWs to be included in organizations’ core operational budgets? (e.g., the base grant of the Bureau of Primary Health Care)
	Next, I will provide you with some scenarios, and I want to get your recommendations or advice on how to respond to the following: An organization has a CHW champion who is invested in ensuring CHW positions are included in the operational budget for an organization. What strategies or recommendations would you provide to this CHW champion as they move forward with this work? An organization is expanding its sources of revenue to ensure the sustainability of CHW positions. What type of funding resources would you recommend? An organization is beginning to plan for its next 5 years. What recommendations would you provide this organization on sustaining its CHW workforce?
	Does your organization have plans to sustain CHWs beyond the life of the CCR program? If so, please describe.
Program partner	Could you share with us what your vision for sustaining CHWs within a partner collaboration should look like?
	As a partner in the CCR program, have you observed collaborations? Has it had a positive impact on strengthening the CHW workforce? If so, how?
	I would like to share some scenarios with you and get your feedback on each of the following: Two organizations have been partnering on a community program that is led by community health workers. They would like to ensure this program continues without a disruption in staffing. What would be your recommendations for needed steps to create a policy or plan for sustainable reimbursement of the CHWs? Two partner organizations have been working on a shared plan for building and sustaining their CHW workforce. What are some essential areas these organizations should be including within their plan?
	Would you like to highlight any examples of challenges that need to be addressed when partners are working on developing strategic plans for sustaining the CHW workforce?
	Could you share examples of strengths or solutions when developing strategic plans for building a sustainable CHW workforce?

### Qualitative analysis

Interviews were recorded, transcribed, qualitatively coded, and analyzed using Dedoose software version 7.0.23 (19). Data analysts used a descriptive/thematic approach with deductive coding (i.e., concept-driven coding) and open coding, with grounded theory overtones of analytic induction of coding and constant comparison as procedures for verifying a theory based on qualitative data (20). This approach was chosen because it places participants at the center of the research question, ensures findings are rooted in the data, and provides a rigorous, iterative framework for data collection, analysis, and coding (21–23). First, we engaged in concept-driven coding, a deductive coding approach whereby researchers create themes from previous studies, research literature, or the interview guide (24). This concept-driven coding included establishing general, *a priori* themes using the interview guide and the Public Health Program Capacity for Sustainability Framework as a guide.

Four independent analysts (AWL, DH, HP, and TG) used the completed codebook to analyze the interviews. All analysts are female-identified, members of minoritized communities with doctoral degrees (e.g., PhD), and were members of the Arizona State University evaluation team, and viewed themselves as CHW allies; one analyst (AWL) is an associate professor. Three analysts (AWL, DH, TG) were trained and seasoned qualitative researchers who provided mentorship in qualitative coding and analysis to the fourth analyst (HP). Two analysts (HP and TG) did not participate in the data collection process and served primarily in analytical roles. Interview transcripts were divided into sections such that each transcript was independently reviewed by two analysts. The analysts read the transcripts and applied codes using the developed codebook.

An iterative analytical process informed by constant comparison was used to examine patterns within and across interviews. This process involved reviewing how codes and themes were applied across transcripts to promote consistency and refine thematic interpretation (20). Analysts independently reviewed each other’s application of codes and themes to ensure consistent application across the interviews. Analysts met to discuss and reconcile any coding discrepancies. Inter-coder agreement reached approximately 90%, reflecting a high level of consistency in coding. The point of data saturation was determined when the coders began to see the same themes emerging across the responses of all three categories of participants. This repetition showed that enough data had been collected. Through ongoing discussions, the research team agreed that reviewing more interviews would not add new insights or change the overall findings. Methodological reflexivity included each analyst’s use of memos as we coded and reflected on those memos, as we discussed any coding discrepancies to ensure we were taking into consideration the nuances of timing and context of the interviews. In addition to the coding memos, analysts reviewed interview field notes to ensure that the manner in which coding occurred took into account each participant and each participant category.

## Results

**Figure 1:**
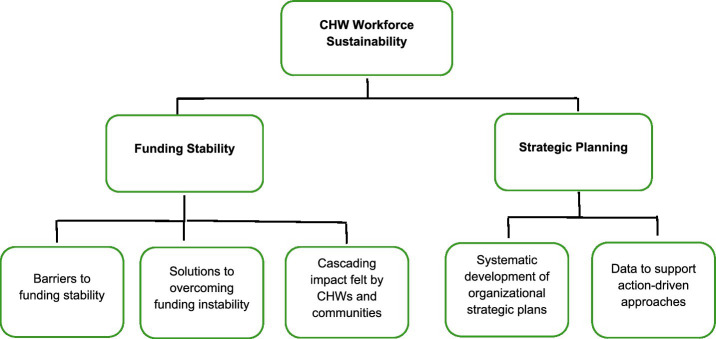
Thematic analysis coding tree.

A total of 27 individuals participated in the interviews. Interview responses coalesced around two major themes guided by the Public Health Program Capacity for Sustainability Framework (i.e., funding stability and strategic planning), and five sub-themes (see Figure 1 for the Coding Tree). Table 2 illustrates the themes as expressed by CHWs, program staff, and program partners, and it provides participant-identified strategies for overcoming sustainability challenges within each of the identified themes and sub-themes.

**Table 2:** Themes, illustrative quotes, and participant-identified strategies to advance the sustainability of the CHW workforce.

Theme/sub-theme	Illustrative quotes	Strategy (ies) to advance the sustainability of the CHW workforce
*Overall funding stability*	Work for me, that would look like working for the Health Department, me working for the local health department. That will look like having my fellow CHWs hired directly through the Health Department. To have a department, a program of CHWs with corporate funded roles through the local health department, which would play a part of longevity, sustainability for their workforce.	I would suggest building, you know, trying to collaborate with other CBOs. And maybe even being a part of a hub design or collaborating with other CBOs, and strategizing on the strengths and the weaknesses of each. What each CBOs brings to the table so that they can work together. To find funding, to apply for funding, to learn how to bill. You know, when they get this funding to learn how to bill. I envision a hub design of all these different entities that can help plan for the next 5 years to sustain CHW work. Bring people to the table, the stakeholders reaching out, having those meetings with key stakeholders who are already doing the work at the local level and trying to align. I recommend aligning your work with what’s going on at the State level, just always being aware of that and building a community around it so that you know the foundation. The infrastructure is there to sustain the CHW work. I think a lot of times people do not do that. Organizations do not do that, or CBOs do not know how to do that, or do not know where to start or they just do not have the manpower to take on the charge or the initiative. So just being a part of organizations could be key to planning out the next 5 years, and of where you can sustain, you know the CHW work betting off each other’s strengths and weaknesses, and just coming together and creating a master plan will be my recommendation.
*Funding stability: barriers to funding stability*	Our grant ran out. Officially, I think we finished using a lot of the funds in 2023 because they were exhausted, but we have also persevered. We are currently exploring billing through [participant’s state medical assistance program] and Medicaid, but from what I see, it is a bit demanding. It does affect the fact that we are used to always focusing on the well-being of people and our patients. I think it affects our effectiveness. It affects our ability to be impactful. Yes, it really affects [CHWs] a lot.	I think it’s like with the nature of CHWs, right? Because not that we are just grant funded. It’s like a lot of these resources are also grant-funded. So I think more stability in programs and more openness about qualifications. I think sometimes it’s good when things have specializations because then it helps narrow it down when you are searching for it. But I think at the same time, like, there are still communities that are forgotten. For example, I work with a lot of the migrant immigrant community. And just because they do not match a certain qualification, then it’s like this whole resource that would really benefit them is closed off to them. So, I think, you know, the part of being a CHW is helping those that are usually, like [for lack of] a better word, ignored or excluded from proper care. And I think in a lot of ways they are missing the mark, it’s not hitting the totality of communities that I serve. I know that many people, many CHWs do [deal with this] across the country. Because it’s not just a local issue but a national issue.
*Funding stability: solutions to overcoming funding instability*	Having these coordinated care organizations and Medicare allow for that. And include those billing codes would be my biggest thing. I think right now they would not have restrictions on being able to serve this bucket of people for this amount of time and would not be narrowly confined to kind of medical Medicaid standards for what you can bill for. But the funding would be there just to cover their fringe and their salary, and let them do what they know the community needs done. That would be my vision, that they are just fully funded. And they get to decide because they know what the community needs and they get to do the work, and I do not have to micromanage them and make sure that they are fitting into defined buckets, and that they are only serving people that are eligible from this funding stream or that funding stream.	In an ideal world, CHWs and the associations are at the table when people are creating these funding opportunities. We’ve seen several grants that maybe were not so great that were maybe aimed at supporting and expanding the [CHW] profession, but made it honestly more complicated. And so it would be really ideal if CHWs were a part of that from the beginning, so that the funding actually did what it was supposed to do.
*Funding stability: impact felt by CHWs and communities*	And even when I say I have some funds, and I can help so we are working toward getting some more supply closets like household items and writing that into our grants. But the real needs are food which most Federal grants will not let us purchase and culturally appropriate food, because in our populations we are working with a lot of refugees and immigrants. So food banks aren’t always giving out the food, or we have a partnership with [partner organization]. But what we are giving them is not what they’ll eat in some cases. So I think it really would be helpful if we had the concrete resources. And then my community health workers would always say that right now people are just really struggling financially. So help with assistance with rent or those real needs. That’s what people are really struggling with, and we do not have any way to do that.	The other thing that was really helpful was thinking about concrete resources is that through this grant we were able to give essential needs cards like gift cards which were available through the [partner organization], and those were immensely helpful. Because again, my community health workers tend to just stop at Walmart and buy the thing when the client needs it. But when we were able to have that supportive funding, and at my agency we are not supposed to use grant funds on gift cards. And there’s all these restrictions and but they are helpful. And they we call them essential needs cards, and that’s what they were for essential needs. And so I think, any way that we can kind of even support even if it seems small, but it’s big on the day to day level that community health worker can take the client and go buy the diapers that they need or make sure that new mom has some basic baby supplies when she gets home from the hospital or they can go to the grocery store. So that’s another piece that I’m usually asking for. If it’s donated, I can have my community health workers document it. And it’s okay. We’re allowed to get that out. A lot of grants and Federal grants exclude purchasing food and gift cards. We’re missing kind of the day to day needs that a lot of clients need.
*Strategic planning: systematic development of organizational strategic plans*	When I think about sustainability, I think of that awareness of who community health workers are. There needs to be a plan like almost a marketing plan that speaks to community health workers. It is just like when they are talking, when they talk about different products. It’s so in your face that when you think about it, you automatically go to that particular product. So when you think about having improved health outcomes, community health workers should be top of mind. So I’m even going to include a marketing campaign that effectively speaks to the value of community health workers and what they bring to organizations, their return on investment. Some programs have said, for every dollar spent they have saved 8 dollars by having community health workers be a part of it. Therefore, that awareness should be top of mind.	If an organization is a CHW champion the first thing they have to do before they include any operational budget, is to find out what is a living wage or a proper wage, how much that costs and view it against the organization’s current earning scale because that is important. The CHWs positions requires training budgets. [The organization] has to think about the types of training: are they for new skills, new topic proficiency, or to move the CHW to the next tier within the CHW profession? In our case there were one or two opportunities where some of our staff wanted to upscale with CHWs. So we have. How? What does it cost to train, whether from entry level or whether you are upskilling staff that is, in the system. So all of that is so you look at as a strategy before you put it in the budget.
*Strategic planning: data to support action-driven approaches*	I’ll give an example. Somebody asked me last week. They said with therapists, they only have to spend physically 16 min with the clients face-to-face or on the phone. Virtually in order to actually bill for that 30 min increment. Is that the same with CHWs? We went and asked Medicaid, and they said, Yes. But that’s not in the guide somewhere. If you are not familiar with these processes. If you have not gone through this, and especially if you are maybe totally new to this Medicaid world, right? You’re a CHW, [you] did not sign up [to navigate Medicaid] that wasn’t what you were thinking about when you became a CHW. But now you are inundated with this. You do not know these unwritten rules of what you are supposed to do or not do. Unless you ask.	At [University] we also had –we have tried to integrate this model with some subsequent grants, academic grants, research grants where we are trying to incorporate citizen scientists and community-based organizations into our evaluation and assessment for those research projects. So, we are trying to get community-based input, and so we would.

These are direct quotes provided by the participants. The use of brackets is to deidentify the participant’s identity or to add clarity to the quote.

### Funding stability

The funding stability theme coalesced around three sub-themes: (1) *barriers to funding stability*, (2) *solutions to overcoming funding instability*, and (3) the *cascading impact felt by CHWs and communities*. Respondents from all three interview groups discussed the overall theme of funding stability by describing the challenges with current funding streams, and most acknowledged that strategies to overcome these barriers included multiple sources of funding and a commitment by organizations to incorporate CHW positions within the existing “corporate” or operational budget to ensure long-term CHW positions. One CHW stated,

[CHWs need to be] […] a consistent part of the corporate budget […] consistency, so that we don’t have the anxiety of having to lay off staff or cut positions or cut services to the community.

Other participants acknowledged that billing Medicaid or Medicare to fully fund CHW positions was not a sustainable solution and was described as a deficient model for CHW sustainability. One program staff member and CHW supervisor remarked,

It [billing Medicaid or Medicare alone] wouldn’t add up to [the necessary] salary; it would not be a way for us to hire more CHWs. We’re studying it as a sustainability option, knowing that there’s not always going to be grants, and even looking at [Participant’s state] now and saying you can bill Medicaid. I’m trying to advocate that really what they [CHWs] do is much more than what’s [currently] billable.

An understanding of the challenges and limitations of how CHWs are currently paid was evident, and it seemed to occupy the mindset of not only CHWs but also of program staff and program partners. Notably, participants mentioned that a variety of funding mechanisms were needed to work in concert. Participants provided specific recommendations regarding how their organizations dealt with these challenges by combining Medicaid reimbursement, collaboration with partner organizations to cover CHW salaries and positions, and adding CHW positions to the operating budget of their organizations (highlighted in Table 2). In the literature, this method is referred to as a *braided funding mechanism;* braiding is a financing approach that combines different funding sources for a program or initiative but tracks funds individually for reporting purposes (25).

#### Barriers to funding stability

Many organizations with CHW positions acknowledge that these positions are often short-term or contingent on grant funding, and participants reflected on the impact that unstable or short-term CHW positions have had on the communities being served. One CHW stated,

We’re also contracted workers. And I think [what if it was] more permanent if it wasn’t like when this contract ends, you may or may not get renewed. If you’re working with a client and you don’t know [if you will have the position] and then you have to put them off for a little bit and say ‘well I don’t know how I’m going to be here by next week’. The uncertainty does make it a little bit hard to keep a stable connection.

The lack of certainty surrounding their positions after grant contracts end prevented CHWs from making future commitments with clients and impacted their relationships within communities.

#### Solutions to overcoming funding instability

Participants identified problems with a scarcity of sustainable CHW positions and shared possible solutions to counteract the lack of funding stability (additional solutions are highlighted in Table 2). One CHW supervisor stated,

When they applied for the grant, it was awarded to the Board of Supervisors, [with the understanding] that this would be funded as a fully funded position that would go beyond the grant. All of our positions have been permanent. Part of the conversation in the beginning, when looking at the RFP, was that they had to have a sustainability plan to even apply for a grant, which was very helpful. And it also motivated us and gave us that [bargaining] card. I guess you can say whenever we were speaking to supervisors or any higher-ups within their organization let[ting] them know that sustainability was a big aspect of this grant and that our overall goal was to create more CHW jobs and that they [CHW jobs] wouldn’t end by the end of the grant, that they would continue.

This quote speaks to the level of CHW champions truly understanding the complexity of ensuring CHW sustainability by adding retention requirements and being thoughtful throughout the duration of seeking funding opportunities such as reviewing the request for proposals (RFPs), to drafting the grant proposal with the inclusion of a CHW sustainability plan to ensure that the position would not rely on grant funding but would be incorporated as a position within the organization’s operating budget once the grant had ended. Other themes related to sustainability solutions highlighted the importance of the inclusion of CHW voices within organizations, the inclusion of CHWs in creating request for proposal calls, and the need to find solutions to limitations in Medicaid and Medicare billing restrictions for CHW services (see Table 2).

#### Cascading impact felt by CHWs and communities

The participants reflected on their perspectives on how the lack of sustainable CHW positions affected CHWs, their organizations, and the communities they serve. One CHW stated,

It affects me 100%. So much so that I’m always looking for another, more stable career. I like what I do, I love what I do, but I feel that it is not sustainable over time to live on grants and wait to see what will happen. [Often the solution is] to place me in this position that I don’t like, but to keep me in that position they change my departments to be able to keep me there. I am totally grateful, 1,000%, because it is wonderful to still be there. It’s very destabilizing to be changing your mind[set], ‘Now I don’t work with these types of people, now I work with this population. Now I’m working on this one’.

In addition to the effects CHWs experienced directly from the lack of employment stability, participants worried about the lasting, detrimental impacts that not having the CHW positions had on the communities they were serving. One program staff member stated, “It’s damaging to a community to put services in and then take them back and put services in and take them back.” One CHW participant provided a poignant example,

Because as an outreach worker, it’s like a spider web. I’m out there at these different agencies, helping people, the folks know where to come to see me. If that goes away, the whole house of cards falls. I know that sounds dramatic, but because people know where to come to interface with me and I’m at these different places, what they can count on, would go away. I’ve been concerned. I’ve been thinking about this. It’s like, am I on a ship that’s sinking or what’s happening?

The study participants were very aware of the limitations of the CHW position due to the constraints put forth by the organization, funding sources, or both, and the overall cascading impact these constraints had on fully assisting community members in need.

### Strategic planning

The respondents’ description of strategic planning could be placed within two prominent sub-themes: (1) the *systematic development of organizational strategic plans,* and (2) *the inclusion of data to support action-driven approaches* for ensuring sustainability of the CHW workforce within their organization and the expansion to include partners.

#### Systematic development of organizational strategic plans

To be systematic in developing organizational strategic plans, one program partner manager stated,

You should align any budget allocations with the organization’s strategic goals for service. Their business operations, usually in healthcare, have a service goal and a service strategy. So align any budgetary operations with that. It’s always good if you can identify a case study or a working example you can use as baseline. In that case, invite or encourage some sort of dialogue or discourse with a health system where the CHW integration or CHW introduction has proved fruitful and successful and have that create that opportunity for dialogue with the power [players] on both sides.

As evident in this quote, CHW program managers must be strategic, including aligning program goals with organizational strategic goals and using anecdotal success stories to help illustrate how CHWs are assets to organizations. Participants also acknowledged that having a long-term approach to sustaining CHW positions included integrating CHWs at all levels of organizational planning. This recommendation includes creating strategic plans before writing grant proposals and having a sustainability plan integrated into the strategic plan to avoid the CHW sense of instability within their position.

#### Data to support action-driven approaches

In addition, providing both qualitative and quantitative data is essential for process, impact, and outcome measures that can be shared as goals and objectives during planning and evaluation. Having data to support action-driven approaches was emphasized by a project manager,

This is data driven. […] You can show where CHW intervention has led to less money spent on illness and care, where CHW intervention has resulted in other determinants being improved for either one population or for a community. […] You have to get the data on the examples for that to work.

In addition to the importance of collecting data to support the impact of CHW work, CHWs acknowledged the need for data in their day-to-day navigation of billing for services. Data on the rules and regulations for reporting billable hours to Medicaid and Medicare, and how to navigate those complex systems, are described in Table 2.

## Discussion

This qualitative, participatory case study describes the challenges and possible solutions for CHW sustainability within organizations and care teams. By utilizing a participatory evaluation approach and including CHWs as decision-makers in all aspects of the research design, our team generated study questions and findings that were driven by the CHW community and therefore relevant to this important public health workforce. Indeed, our study findings demonstrate that participatory action is not only essential for conducting research that is meaningful and relevant, but it is also necessary for developing and implementing solutions to CHW workforce sustainability that have the potential to be viable and effective.

The centering of CHWs’ and CHW champions’ perspectives provides a poignant example of the importance of CHWs having stability within their organizations to provide the best care and support to the communities they serve. The work conducted by CHWs is time-consuming and complex, often requiring that CHWs provide wraparound services; yet the instability of their grant-funded positions interferes with their ability to work most effectively within their communities.

Our findings regarding the impact of the lack of sustainability in CHW positions on the community align with the challenges highlighted by Saint Onge and Brook (12). These authors discussed three forms of capital, economic, cultural, and social, which are individual resources used to situate one’s position in the field. Specifically, the authors found that CHWs had cultural and bonding (social) capital, which created and maintained trusting relationships between CHWs and the communities they serve. This capital did not translate into the organization’s (economic) capital and reinforced structural inequalities when social capital was restricted, and CHWs had limited access to economic and institutional cultural capital within the healthcare system.

Our findings are also consistent with other studies [e.g., (1, 15)] that stated that securing sustainable financing for CHWs remains a key objective among stakeholders. Barbero et al. (1) noted that in their review of statewide CHW workforce initiatives, many discussed Medicaid financing models, but despite being widely promoted as a pathway to sustainability, the inclusion of CHWs within Medicaid State Plans was only found in one-fifth of U.S. states. Additionally, our participants were quick to point out that the current Medicare and Medicaid reimbursement system would not fully cover the salary for a CHW position and would not assist in growing the CHW workforce as it currently functions. This finding adds evidence to the call for Medicaid administrators to leverage existing channels of communication with Centers for Medicaid and Medicare (CMS) officials to advance the development of metrics that more accurately capture the CHW processes and outcomes, including metrics related to the social determinants of health, and appropriate methodology for measuring CHW integration within healthcare systems and teams (15).

The 2018 WHO guidelines for CHW programming emphasized that achieving large-scale CHW initiatives requires long-term, dedicated financing infrastructure, and that relying on a shoestring budget is likely to yield disappointing outcomes (26). Our findings highlight the need for researchers to continue collecting evaluation and implementation data to demonstrate the effectiveness of CHW programs and use this data to advocate for and gather the political support to sustain the CHW workforce. As Perry et al. (2) stated,

We are long past due for a shift in our understanding of CHW programmes, from viewing them as a temporary and underfunded afterthought, to seeing them as an integral component of optimally functioning health systems anywhere in the world, including high-income countries and [low- and middle-income countries] LMICs. (p. 14).

Our findings suggest that the current financial and political infrastructure in the United States has created barriers to sustaining the CHW workforce within our health systems. The healthcare system prioritizes insufficient state and federal government resources that focus on vertical programming (e.g., disease-controlled and hospital-centric programming) rather than horizontal programming (e.g., integrated community-based primary healthcare services provided by CHWs) (26).

It is important to note the limitations of the current study. As a qualitative, participatory case study involving a subset of CCR recipient organizations, the findings are not intended to be generalizable. Rather, they are grounded in the specific contexts and experiences of CCR-supported sites in the United States. The insights presented may be transferable to similar settings or programs, particularly those navigating comparable funding and policy environments, but should be interpreted with consideration of contextual differences, including those present in low- and middle-income country (LMIC) settings and global CHW initiatives. Additionally, the study team elected not to obtain demographic data due to the small CHW sample sizes at some of the recipient sites, which limited our ability to explore how identity factors such as ethnicity and gender might influence experiences of sustainability. Second, although all interviewers were trained and used the same set of questions, it is impossible to ensure each interview was identical due to the nature of human conversation. Additionally, qualitative analysis brings a level of bias, but it is important to note that our team utilized a team approach to check and assess coding alignment, included memos to identify and address potential researcher biases, and held team meetings to discuss any disagreements about coding. Third, interviews were conducted during the final year of a 3-year grant period when sites were focused on securing funding to sustain their programs beyond the life of CCR or were planning the culmination of their programs; this timing may have impacted which individuals were willing to participate in interviews. At the same time, the timing may have impacted the topic of sustainability that was at the forefront of participants’ minds. Finally, we presented on two of the four domains from the Public Health Program Capacity for Sustainability Framework, which may limit the ability of this study to capture other elements associated with CHW sustainability.

Future studies should consider including a return on investment component. As indicated by one respondent (Table 2), knowing the effectiveness of CHWs can be utilized as a marketing strategy and data source to continue growing the CHW workforce. In addition, knowing the amount of time and effort CHWs spend on ensuring a patient is navigating through the healthcare system would also provide organizations with concrete data on whether Medicaid payment allocation is consistent with the needs of the community. Additional research using return on investment methods, particularly those that consider measures of health-related service issues (e.g., education, housing, food, transportation, reentry, etc.) for individuals and families, is necessary to advocate for the value of CHWs (14). CHW-driven solutions (Table 2) demonstrate that the CHW workforce recognizes the challenges but is keenly aware of the steps that need to be taken to achieve CHW sustainability within organizations. Finally, it is important to recognize the impact of using mixed methods to provide nuanced descriptions of the mechanisms or structural processes of CHW-built trusting relationships, which in turn lead to improvements in client adherence to treatment, improved health outcomes, and reduced health disparities (27).

## Data Availability

The datasets presented in this article are not readily available because we are currently analyzing the data for this project and have data restrictions until the completion of data analyses by the investigative team. Requests to access the datasets should be directed to Ada Wilkinson-Lee, adaw@arizona.edu.
